# Primary Processes of Depolymerization of Lignin Dispersed into Gas Phase

**DOI:** 10.1021/acs.energyfuels.4c06278

**Published:** 2025-05-05

**Authors:** Marwan Y. Rezk, Mohamad Barekati-Goudarzi, Divine Nde, Dorin Boldor, Slawomir Lomnicki, Stephania Cormier, Lavrent Khachatryan

**Affiliations:** † Department of Biological and Agricultural Engineering, Louisiana State University and LSU AgCenter, Baton Rouge, Louisiana 70803, United States; ‡ Department of Chemistry, Louisiana State University, Baton Rouge, Louisiana 70803, United States; § Department of Environmental Sciences, Louisiana State University, Baton Rouge, Louisiana 70803, United States; ∥ Department of Biological Sciences, LSU Superfund Research Program and Pennington Biomedical Research Center, Baton Rouge, Louisiana 70808, United States

## Abstract

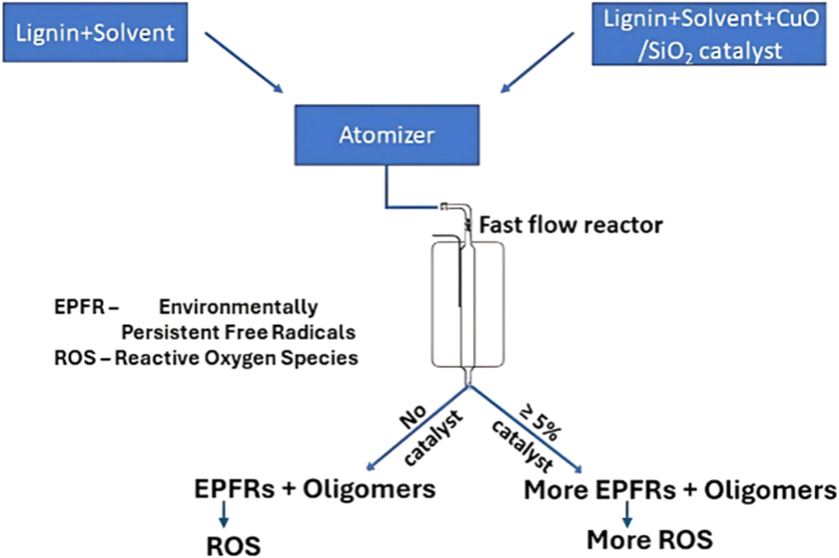

The primary depolymerization processes of hydrolytic lignin (HL) are examined, focusing on the formation of intermediate oligomers and bulky environmentally persistent free radicals (EPFRs). Fragmentation of HL was conducted in a continuous atomization (CA) fast flow reactor, where HL, dissolved in a 9:1 acetone-to-water solution, was dispersed. Results indicated that HL fragmentation occurs significantly faster in the gas phase in comparison to the literature rate of formation of major biofuel-phenolic compounds. In other words, the formation of phenolic compounds occurs at much lower rate constants being the limiting stage for lignin depolymerization. The critical role of surface associated reactions for formation of biofuel compounds developed in our previous work was highlighted. Using spin trapping with electron paramagnetic resonance (EPR) spectroscopy, it was shown that intermediate EPFRs, as hydroxyl radical generators, may act as biologically active intermediates in aqueous environments relevant to anthropogenic activities, wildfires, tobacco smoke, and other combustion processes. The addition of a highly hydroxylated 5% CuO/SiO_2_ catalyst at concentrations of 1–3% (relative to an initial lignin concentration of 1 g/L in a 9:1 acetone-to-water mixture) did not significantly alter EPFR yields. However, an increasing trend in EPFR yield was observed with catalyst concentrations at 5%. A mechanistic scheme for the formation of CuO-surface-associated EPFRs is discussed.

## 1 Introduction

The development of successful strategies for lignin conversion into various value-added products requires the design of effective depolymerization protocols. Most research on lignin depolymerization has focused on batch-mode processing. However, the detailed mechanism of lignin pyrolysis in conventional reactors remains unclear, largely due to its complex structure and random composition.^1−4^ Mass transfer limitations on polar and higher molecular mass compounds, the influence of reaction conditions on the yields and kinetics for nascent compounds derived from lignin pyrolysis, and the unknown nature of reactions taking place in the solid phase limit the understanding and elaboration of lignin pyrolysis mechanisms.^2,5^ In addition, the substantial intermolecular interaction between end groups of lignin macromolecules causes an artificial delay in product release that is unrelated to the pyrolysis mechanism. To eliminate most of these interferences that naturally occur during solid-phase lignin pyrolysis, several nonconventional, contactless reactors were designed in the Chemistry department at LSU^6−9^ for depolymerization of lignin. This enabled our group to elucidate the primary processes and genuine pathways involved in lignin depolymerization using a continuous-wave (CW)-CO_2_ laser powered homogeneous pyrolysis, LPHP flow reactor^7,10^ and a continuous atomization (CA) flow reactor.^6,8,9^ In both reactors, HL lignin, dissolved in acetone: water (9:1) solution, was dispersed into the gas phase using an atomizer. The short residence time (on the order of milliseconds), low mass delivery through the flow reactor, significantly minimize the surface effects typical for the conventional batch reactors, and rapid quenching of reaction products effectively suppress unwanted reactions, particularly secondary repolymerizations. In these works, the reaction intermediate fractions collected at the end of CA and subjected to electron paramagnetic resonance (EPR) analysis in the large range of frequencies from conventional X band (9.7 GHz)^6^ to high-field EPR (HF-EPR, 413 GHz),^9^ revealing the existence of carbon and oxygen-centered stable radicals. Ultimately, we proposed that the primary mechanism for lignin depolymerization at high temperature (500 °C) involves the fragmentation of HL through the formation of intermediate oligomer radicals and other neutral oligomer fragments which then produce a variety of organic compounds in secondary reactions.^6,8,10^ It is remarkable, that these processes are nearly free of phenolics.^8^

Radicals trapped from HL pyrolysis in the CA reactor have been proposed as a new category of metal-free environmentally persistent free radicals (EPFRs), referred to as bio-EPFRs.^9^ Estimated lifetimes of the two radical groups of bio-EPFRs from lignin gas-phase pyrolysis were 33 and 143 h, respectively, which indicate their persistent nature and the associated negative effects in the environment.^9^ It is believed that bio-EPFRs are stabilized within the lignin matrix in a metal-free environment, like surface-bound EPFRs in biochars reported in previous studies.^11−13^

Generally, surface-bound EPFRs are formed through the interaction of chlorinated or hydroxylated aromatic compounds during incomplete combustion processes in the presence of transition metal oxides^14^ with long lifetimes and, exhibit hazardous effects on biological systems.^15,16^ Extensive reviews on this phenomenon have been widely documented in the literature.^17−20^ The formation of intermediate radicals in the presence of transition metal oxide catalysts plays a crucial role in macromolecules like lignin, which structurally resembles aromatic hydrocarbons such as phenol, catechol, and hydroquinone derivatives.

The current research aims to clarify the kinetic behavior of oligomers, and the behavior of bulky persistent free radicals (EPFRs) generated during the homogeneous pyrolysis of HL in the gas phase across a broad temperature range, (450–550 °C). It presents, for the first time to our knowledge, the performance as precursors for the generation of hazardous reactive oxygen species (ROS), such as hydroxyl radicals, in aqueous environments. Additionally, the study aims to fill a critical knowledge gap in the understanding of the catalytic effect of a specially prepared CuO/SiO_2_ catalyst on the homogeneous and heterogeneous conversion of HL. The details of the CuO/SiO_2_ catalyst synthesis and characterization are reported in our previous publication^21^

The Doehlert design^22^ was used to determine the conditions that produce highest yields of EPFRs from the pyrolysis of HL in fast flow CA reactor in the temperature region from 400 to 550 °C which were then employed to produce EPFRs for ROS studies A detailed mechanistic interpretation is provided for the catalytic transformation of HL in the presence of the catalyst, drawing from extensive research on the formation of EPFRs on “transition metal oxide (CuO)” surfaces in the presence of 2-chlorophenol and catechol, which serve as models for lignin compounds.

## 2 Experimental Section

### 2.1 Materials

HL is a dark brown colored powder purchased from Sigma-Aldrich Inc. (catalog number 37–107–6, discontinued). N_2_ (99.9999%, from Airgas), acetone from Millipore Sigma with purity ≥99.5, high-purity 5,5-dimethyl-1-pyrroline-*N*-oxide (DMPO,99%+) from DOJINDO laboratories, hydrogen peroxide from Fluka (Assay, 30%), 0.01 M phosphate-buffered saline (PBS, NaCl 0.138 M, KCl 0.0027 M), and copper nitrate hemipentahydrate (99.9+%) were purchased from Sigma-Aldrich, and Cab-O-Sil from Cabot (EH-5, 99+%).

### 2.2 Methods

#### 2.2.1 Fast Flow Continuous Atomization (CA) Reactor

The details of the CA experimental reactor are available in our laboratory’s previous publications.^6,8^ The schematic picture of the CA reactor is depicted in Figure S1 (Supporting Information). HL was dissolved in acetone/deionized water (DIW) (v/v of 9:1) mixture in a concentration range of (1–5) g/L. The solution was stirred for 5 min followed by stirring for 2 min and further sonication for 10–15 min to ensure complete dissolution. The solution was loaded into a syringe (Hamilton, 25 mL, 23.03 mm d) and introduced via *KD scientific* syringe pump at a constant flow rate of 7.5 mL/h to a constant output TSI 3076 atomizer. The atomizer had an inlet supply of high-purity nitrogen (99.999%) gas that was regulated with a mass flowmeter (ALICAT Scientific, MCR-series). “Atomized” lignin in the gas phase is then introduced to the CA reactor. The products of the depolymerization process were collected on a preweighed Cambridge filter at the bottom of the reactor. The lignin recovery was evaluated based on the difference of mass of the Cambridge filter to be 80–85% from the top of the reactor. The collected products of atomization were subjected to EPR analyses.

The details of EPR, electrospray ionization mass spectrometry (ESI MS), and spin trapping methods are presented in the Supporting Information.

### 2.3 Doehlert Design^22^

We used Response Surface Methodology (RSM) and Doehlert’s design^22^ to quantitatively evaluate the dependence of the concentration of free radicals from depolymerization of lignin under different operating conditions of temperature and residence time in the CA reactor. Consequently, RSM was used to find the most optimal conditions for the highest concentration of free radical generation which is critical for spin-trapping experiments (*vide infra*).

The use of the Doehlert experimental design avoids the several drawbacks associated with the univariate optimization technique including its inability to predict the interaction effects between variables. This design was planned and executed to investigate the combined or interaction effects between residence time (0.1–0.6 s) and temperature (400–550 °C). Herein, we define the residence time as the time lignin takes from the inlet to the outlet of the reactor. Doehlert’s design allows the use of the least number of observations to estimate the behavior of the experimental system compared to the other known designs. The lignin depolymerization process was assumed to follow a second-order polynomial as shown in eq 1. β represents the model coefficients, *X*_1_ is the residence time and *X*_2_ is the depolymerization temperature. The model coefficients were determined by regression and the predicted results were compared to those obtained from experiments to validate the results. Optimum points were obtained by maximizing the equation on the JMP software. To verify the optimum points, separate experiments were performed under calculated optimum points and the results obtained were compared with the calculated ones. The degree of agreement between experimental and calculated results and *R*^2^ values obtained from regression analysis were used to validate the model within the boundaries of the experimental conditions studied in this work.
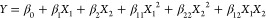
1

The experiments, depending on conditions, were run in duplicates or triplicates.

## 3 Experimental Results and Discussion

### 3.1 Neutral Oligomers from CA Reactor at 500 °C

**Figure 1:**
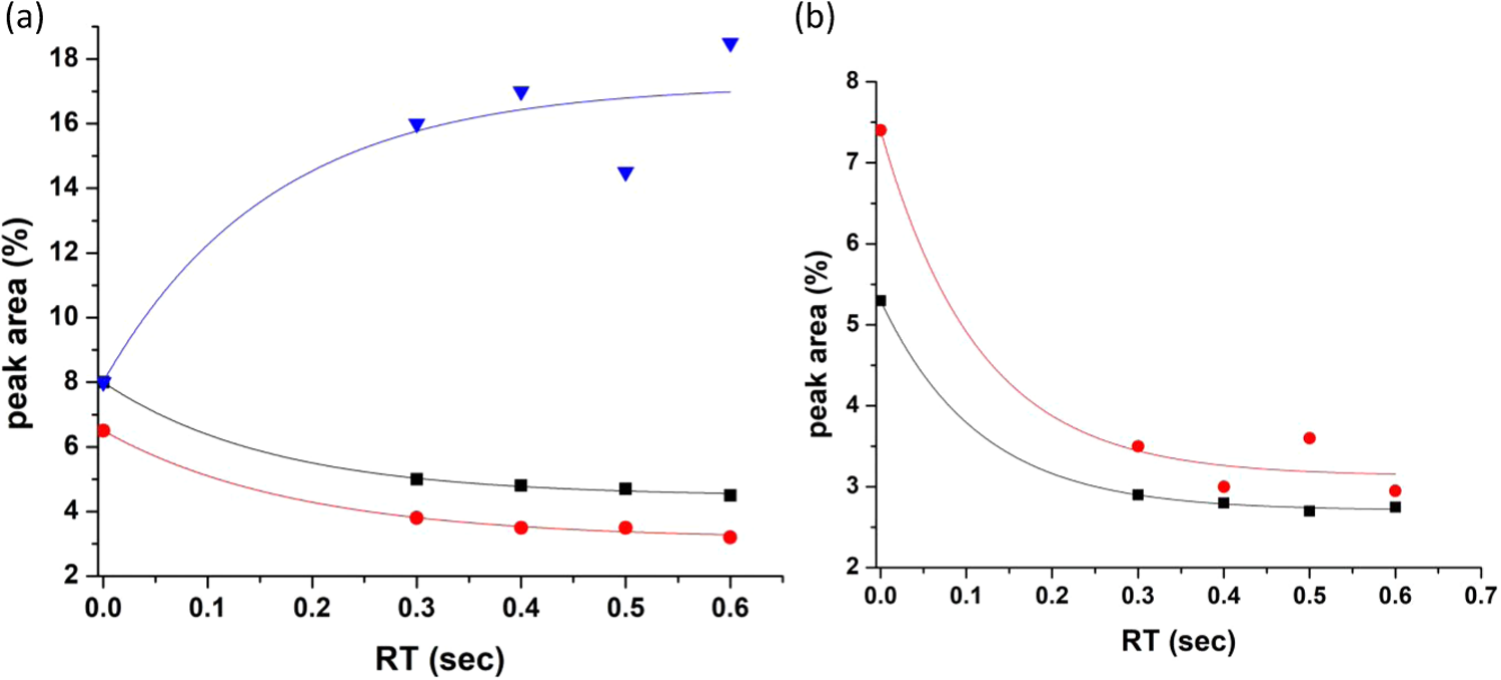
Time dependence of the yields of oligomers grouped in the range 100–250 Da (light fraction in blue triangles)—Decomposition profile of grouped oligomers; (a) 850–1000 Da (black boxes), 1000–1150 Da (red circles), (b) 1150–1300 Da (black boxes) and 1300–1600 Da (red circles). Each experimental point is an average of triplicate measurements.

Neutral oligomers were collected from the CA reactor and analyzed using High-Performance Liquid Chromatography (HPLC) coupled with accurate mass electrospray ionization (ESI) mass spectrometry (Supporting Information, section 2). The oligomers were grouped in the range; 100–250; 250–400; 400–550; 550–700; and 850–1000 Da. The representative graph is depicted in Figure 1. Note that the HL by itself contains all the oligomers in the range presented (from 100 to 250 to 1000 Da).^23^ Triplicate injections of HL were performed with this set of experiments. The fraction 100–250 Da was not specifically identified, but it is assumed that this group presents some mixture of monomers and dimers. Other groups are most probably a mixture of dimers, trimers, etc. Remarkably, oligomers from all other groups (except the first group, 100–250 Da) are slowly decomposed (Figure 1, oligomer group 850–1000 Da). At the same time, the yields of the products in the group 100–250 Da slowly increase with the residence time (Figure 1) indicating the occurrence of dimerization processes.^24^ The following results demonstrate the identification of various oligomer groups, up to 1600 Da, across a broad temperature range of 450 to 550 °C in the CA reactor (Section 3.2).

### 3.2 Kinetic Behavior of Oligomers as Intermediates from HL Homogeneous Pyrolysis

Kinetic measurements were conducted to examine the yield dependence of oligomers (spanning a broad molecular weight range, up to 1600 Da) from HL pyrolysis as a function of residence time in the CA reactor. Oligomers with molecular weight higher than 250 Da were slowly decomposed vs residence time from 0.1 to 0.6 s in the CA reactor, Figure 1(a). During pyrolysis of HL dispersed in the gas phase, the initial macromolecule (or some oligomer fractions widely present in the initial lignin^23^) breaks down to smaller fragments in primary processes (at less than 10% conversion.^6,8^), Figure 1. The pseudo-first-order rate constants from these kinetic curves of decomposition of certain oligomer groups vs residence time can be deduced, Table 1. The rate constants are in the range of 1.11–1.77 s^–1^ abstracted from the equation for the monomolecular reaction (eq 2)
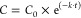
2where *C* and *C*_0_ are the current and initial concentration of oligomers at a given time, respectively and *k* is the pseudo-first-order rate constant in s^–1^. This process is also accompanied by the formation of oligomer radicals shown elsewhere.^24^

**Table 1:** Pseudo-First-Order Reaction Rate Constants Derived from Figure 1 for Each Group of Oligomers (at 500 °C)

	mol weight of oligomers (Da)			
residence time, s	850–1000	1000–1150	1150–1300	1300–1600
0	7.9	6.5	5.3	7.4
0.3	5	3.7	2.9	3.5
0.4	4.7	3.5	2.7	3
0.5	4.6	3.4	2.7	3.5
0.6	4.5	3.2	2.6	2.9
rate constants, s^–1^	1.108	1.356	1.413	1.77

**Scheme 1:**
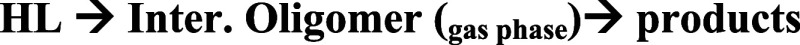


No detectable amounts of simple phenolics were observed. All these discoveries affirm the predominant pathway in the decomposition of lignin macromolecules, which primarily leads to the formation of intermediate neutral oligomers (**Inter. Oligomer**, Scheme 1), including bulky radicals. Subsequent transformations of these intermediates can result in the generation of smaller biofuel molecules, such as the phenolic compounds commonly observed in conventional reactors.

Scheme 1 appears to represent the primary pathway for depolymerization of lignin. As mentioned above, there is a large discrepancy between measured homogeneous rate constants (1.1–1.77 s^–1^, this work, Table 1) and the rate constants for lignin fast pyrolysis derived from batch pyrolysis of lignin using various empirical models (0.14–0.031 s^–1^) reported in ref (25) at the same 500 °C. These significant disparities in decomposition rate constants (an average of more than one order difference), highlight the stark contrast between the pyrolysis processes of HL when dispersed in the gas phase and the equivalent processes in a batch reactor.

Our research demonstrates that lignin undergoes rapid breakdown into intermediate neutral oligomers (radicals) with high-rate decomposition constants. However, it becomes evident that the subsequent conversion of these intermediates into phenolics or other small organic compounds does not occur in the homogeneous gas phase. The introduction of any heterogeneous phase (i.e., solid surfaces) into the reactor promptly leads to the production of phenolic-type end products.^8^ This discovery is significant, as it indicates that lignin macromolecules decompose into intermediates with high-rate constants (approximately 1–2 s^–1^) in the gas phase. Yet, the formation of phenolic compounds occurs at much lower rate constants (approximately 10^–1^–10^–2^ s^–1^ at 500 °C^25^), becoming the limiting stage for lignin depolymerization.

**Scheme 2:**
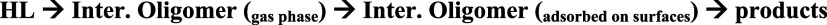


This phenomenon is likely taking place on the surfaces of either the solid lignin phase (in a batch reactor) or, in the case of gas-phase reactions in the presence of solid substrates like metals (catalysts), where these surfaces may greatly enhance the formation of phenolic-type compounds.^8^ This implies that the rate-determining step for the formation of phenolics involves a heterogeneous pathway (Scheme 2) where the decomposition of lignin intermediate oligomers adsorbed on a surface (or formed directly on the surface of the lignin matrix) plays a pivotal role.

This represents an essential development in our understanding of, and potential control over, the production of phenolics as key constituents of biofuels and confirms the hypothesis developed in reference.^8^ The reaction pathway (Scheme 2) is most likely to occur readily on the surface, either on bulk lignin or on a catalyst in conventional batch reactors. Additionally, Scheme 2 could serve as a new approach for developing a lumped mathematical model to describe the formation of major product groups from lignin pyrolysis (tar, char, and gas), which typically accounts for their direct formation from the lignin macromolecule in batch reactors.^26,27^

### 3.3 EPFRs from Pyrolysis of HL and Some Model Compounds on the Surface of a Catalyst CuO/SiO_2_

The previous section emphasized that a surface is essential to initiate the decomposition of HL in the gas phase. Notably, significant lignin conversion was observed when steel or copper wool was introduced into the CA reactor.^8^ To further investigate the lignin transformation phenomenon on solid surfaces, a 5% CuO/SiO_2_ catalyst was utilized to study the primary processes of HL adsorption and decomposition. Copper oxide nanoparticles dispersed on silica (SiO_2_) support have been widely used to study the adsorption and subsequent thermal decomposition of various halogenated and hydroxylated aromatic compounds.^11,14,28,29^ The details of the CuO/SiO_2_ catalyst synthesis and characterization are reported in our previous publication.^21^

The key findings from these heterogeneous processes point to the formation of environmentally persistent free radicals (EPFRs), a new class of pollutants that generate biologically harmful reactive oxygen species (ROS) in aqueous environments, such as hydroxyl, superoxide, and peroxyl radicals, along with other reactive molecules like hydrogen peroxide, various peroxides, and per-acids.^30−32^

#### 3.3.1 EPFRs from Model Compounds and Lignin on the Surface of CuO/SiO_2_: Gas-Phase Exposure Chamber

In this section, we will present results on the formation of EPFRs from lignin and model to lignin compounds (2-monochlorophenol, MCP, and catechol, CT) on highly hydroxylated (HH) surfaces of 5% CuO/SiO_2_^21^ at as low as 230 °C temperature. The practical significance of temperature below 250 °C lies also in the fact that the formation of EPFRs or surface-mediated reactions occurs in combustion reactors (such as waste incinerators, coal-fired systems, etc.) within the postcombustion zone. This region is characterized as the cooler zone, with temperatures ranging from approximately 150 to 400 °C. In this temperature range, EPFRs stabilized on metal oxide surfaces remain intact, avoiding destruction that may occur in hotter zones. Specifically, 230 °C serves as an optimal temperature, allowing for the generation of EPFRs in laboratory conditions in sufficient quantities.

For these purposes, a conventional, homemade gas-phase exposure chamber^14^ for the generation of EPFRs from highly volatile (MCP) or less volatile (CT) organic compounds was employed (Supporting Information, Figure S4). EPR spectra of the EPFRs from MCP and CT are depicted in Figure S5(A). These EPFRs are generated at 230 °C (abbreviated MCP230 and CT230 EPFRs) and possess structureless EPR lines. Note that all EPR spectra of EPFRs detected from MCP and CT are very sensitive to the experimental conditions and depending on vacuum quality (for-vacuum—10^–2^ Torr or high vacuum—10^–5^ Torr) different structureless spectra are registered.^14,33−36^

For nonvolatile compounds such as lignin, a mixture of lignin (1%, w) with CuO/SiO_2_ was prepared and heated at different temperatures in the same aforementioned exposure chamber under vacuum. Pyrolysis is a common thermal approach to depolymerize lignin. Investigating the low-temperature (<250 °C) decomposition of lignin is crucial, as the weak ether bonds begin to break at these temperatures.^37,38^ The addition of catalysts (zeolite, transition metals, etc.) during pyrolysis facilitates the cleavage of β–O–4, α–O–4, and C–C bonds of lignin to improve the yield of aromatic compounds.^39^

The EPFRs spectra from the heating of lignin at different temperatures have a similar shape, with *g*-values and Δ*Hp-p* that are different but closely aligned (see caption under Figure S5). Note that the catalyst, pretreated under various conditions—such as impregnation in different polar solvents and subsequent drying—retains its activity in generating EPFRs. Although the similarity in *g* and Δ*H* values alone (Figure S5) is insufficient to fully characterize the nature of these radicals, it is essential for studying other physicochemical aspects of EPFRs formation, particularly in understanding the biological implications of these emerging radical-type pollutants.^33^

### 3.4 Optimization of Generation of EPFRs from HL in Fast-Flow CA Reactor

The optimum conditions for generating high yields of EPFR in a CA reactor were achieved using the Doehlert design (refer to Section 4 in the Supporting Information for details).

The experimental work through this design yielded an increasing concentration of free radicals at *T* = 486 °C and residence time RT = 0.3 s; the initial *g-value* for intrinsic radicals in HL starts at 2.0043 and decreases to 2.0034 as the temperature increases. The aforementioned optimal conditions yielded a concentration of free radicals of 1.26 × 10^18^ spins g^–1^ collected at the end of the CA reactor, with a *g-factor* of 2.0034 and Δ*Hp-p* = 7.14 gauss. These results confirm that the nature of free radicals produced from lignin pyrolysis in the CA reactor shifts from oxygen-centered to a mixture of oxygen and carbon-centered radicals as has been shown in some of our early publications.^9,40^ A similar reduction of *g-values* in pyrolysis processes is a common phenomenon, indicating accelerated deoxygenation during the pyrolysis of different biomass components as the pyrolysis temperature increases.^41,42^

#### 3.4.1 Persistent Free Radicals Generated from the Homogeneous and Heterogeneous Decomposition of HL in CA Reactor

The EPFRs were generated from the pyrolysis of HL in the CA reactor in the absence and presence of the catalyst, CuO/SiO_2_. Highly hydroxylated CuO/SiO_2_ catalyst^21^ mixed with lignin (1 to 5% catalyst, weight) and dissolved in acetone/water (9:1, (v)) was pulverized into a CA reactor. The ratio of the yields of persistent radicals from pyrolysis of HL at homogeneous (in the absence of catalyst, CuO/SiO_2_) and homogeneous/heterogeneous conditions (in the presence of 1–5% (w) catalyst, CuO/SiO_2_) are summarized in Table 2. The yields of EPFRs in the absence of the catalyst were estimated at a defined ratio of 2.59 at 486 °C and a residence time of 0.3 s. While no distinct change was observed in pyrolysis processes at 1 and 3% concentration of highly hydroxylated CuO/SiO_2_ catalyst; a trend of increasing concentration of EPFRs was observed at 5% concentration, Table 2.

**Table 2:** Relative Yields of EPFRs from HL Pyrolysis in CA Reactor at Different Conditions

CuO/SiO_2_ concentration (w %)^a^	0	1	3	5
bottom/top ratio in CA reactor^b^	2.59	2.6	2.5	4.1

a HL dissolved in acetone/water (9:1) was dispersed into CA reactor without and with a catalyst—CuO/SiO_2_. The concentration of intrinsic radicals entering the CA reactor was 4.46 × 10^17^ spins per gram across all cases considered.

b The ratio of EPFRs produced from the reaction, accumulated on the CF at the bottom of the CA reactor, to the initial concentration of intrinsic radicals in HL (abbreviated as Top).

No difference was observed in the EPR spectra when comparing the top and bottom spectra of EPFRs from the pyrolysis of HL at 486 °C, both with and without the CuO/SiO_2_ catalyst. Note the similarity between the EPR spectra of EPFRs derived from lignin model compounds and lignin itself from the exposure chamber at 230 °C (Figure S5A) and HL pyrolysis from CA overlaid in Figure S5B (line 1).

The trend of increasing radical yield in the presence of a catalyst suggests that the hydroxylated units of copper oxide interact with the lignin macromolecule, leading to a slight increase in radical concentration. The mechanism of interaction of transition metals on lignin pyrolysis in general is controversial.^43^ In this review, the authors concluded that the oxides of nickel and iron have a weak effect on promoting the formation of condensable products from lignin pyrolysis, while the oxides of cobalt, manganese, and copper have an inhibitory effect. The alkali metal ions had dramatic catalytic effects. For example, when comparing the catalysis of potassium and copper it was found that potassium plays a leading role in the catalytic pyrolysis process. Alkali metal ions change the bond dissociation energy(BDE) and particularly promote the β–O–4 homolytic reaction, as demonstrated in theoretical calculations,^44^Figure S9 (Supporting Information, section 5).

**Figure 2:**
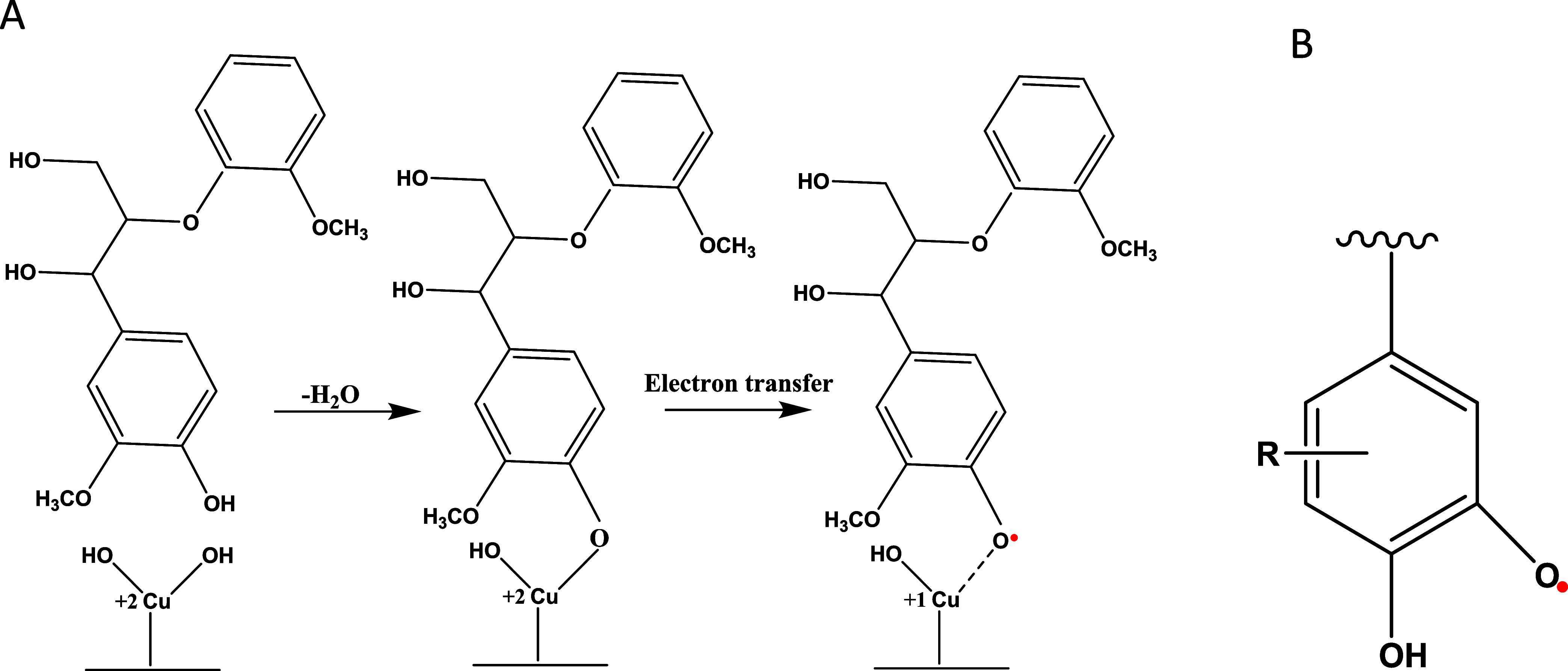
(A) Hypothetic scheme for catalytic formation of EPFRs associated with the highly hydroxylated CuO/SiO_2_ catalyst. (B) Schematic framework for intrinsic radicals in the lignin matrix.

As for transition metals, similar DFT calculations show that among 3d and 4d transition metal ions the catalytic effect follows Fe > Co > Ni > Cu.^43^ In this study, the highly hydroxylated CuO/SiO_2_ catalyst used for HL pyrolysis in the CA reactor may interact not only with the scheme developed for alkali and alkaline earth metals^43^ (Figure S9, Supporting Information) but also through the mechanism of formation of EPFRs reported in previous work.^21^ By consideration of the same dimer **GGE** (β–O–4 model dimer—guaiacylglycerol-β-guaiacyl ether)^43^ as a model of the lignin molecule, the formation of an O-centered radical associated with the highly hydroxylated CuO catalyst on the surface of SiO_2_^9,21^ can be illustrated, Figure 2A. This scenario applies when the lignin macromolecule breaks down into smaller fragments, such as oligomers (Section 3.1), most likely through the cleavage of β–O–4 linkages. The resulting dimers, trimers, and similar products contain aromatic hydroxyl groups—mainly from syringyl (S), guaiacyl (G), and p-hydroxyphenyl (H) units. These groups can interact with the catalyst’s surface OH groups, leading to the formation of large aromatic radicals attached to the catalyst surface, as illustrated in Figure 2A. The substantial decrease in the g-value for EPFRs generated in the CA reactor (from 2.0043 to 2.0034, as detailed in Section 3.4) likely indicates the parallel formation of C-centered radicals, which typically exhibit lower *g*-values (this work and a number of others^6,12,45,46^).

The minimal change in EPFR concentrations during the catalytic transformation of HL in the CA reactor is likely due to either a lower catalyst concentration (a change was observed when 5% catalyst was used, as shown in Table 2) or the low abundance of phenoxy OH groups in the lignin macromolecule.^23,47−49^ This scarcity may hamper the interaction between HL molecules and the catalyst’s surface OH groups (see Figure 2A).

It is worth noting that the radical-chain mechanism of lignin decomposition is one of the pathways for lignin depolymerization, as summarized in several recent publications.^1,43,50^ HL itself contains a high concentration of intrinsic radicals, exceeding 10^17^ spins g^–1^, as reported elsewhere,^6^ aligning with the range of initial concentrations observed in various types of lignin.^51^ An overview of intrinsic radicals, characterized by isotropic g-values ranging from 2.0034 to 2.0047 and identified as semiquinone-type radicals in lignin (refer to Figure 2B) using high-frequency EPR, is provided in references.^50,51^

### 3.5 Oxygen-Centered vs Carbon-Centered Persistent Radicals: Easy Spin Calculations

Radicals from the postpyrolysis of HL consist of paramagnetic fragments of decomposed lignin macromolecules and include newly formed polymerized units with both oxygen and carbon centers, as concluded in a recent publication.^9^ While high-field EPR at 413 GHz did not provide sufficient spectral resolution of these radicals, initial experiments indicate the presence of O-centered radicals within the radical mixtures, which display a high apparent *g-value* of 2.0048 (Supporting Information, Figure S10, black line). These O-centered radicals could include both intrinsic forms and newly formed SQ-type (semiquinone) radicals. C-centered radicals are also assumed to be present in the radical mixture following HL pyrolysis in the CA reactor.^8,9^ To further analyze the radical composition, EasySpin MATLAB software package for spectral simulation^52^ was applied to model the HF EPR (413 GHz) spectrum of EPFRs from HL pyrolysis in the CA reactor (Supporting Information, Figure S10, black line). A reasonable fit between the experimental (black line) and calculated spectra (green line) was achieved by assuming a two-component radical mixture from HL pyrolysis. Notably, the fitting suggested a significant dominance of O-centered radicals over C-centered ones. The confirmation that O-centered radicals are a major component of the radical mixture has motivated spin-trapping experiments, as EPFRs, such as those shown in Figure 2B; it has long been proposed that they actively generate OH radicals.^53,54^

## 4 Spin Trapping

A key objective is to validate the potency of primary EPFRs produced from lignin pyrolysis in the CA reactor as precursors for generating hazardous intermediates, such as hydroxyl radicals. It is important to note that persistent radicals formed from lignin pyrolysis in batch reactors (at high lignin conversion) and stabilized on chars have been extensively reported as precursors for the generation of reactive oxygen species.^12,45,46,55,56^

Herein, we performed spin-trapping experiments using 5,5-dimethyl-1-pyrroline-*N*-oxide (DMPO) to investigate the generation of longer lifetime^57−60^ DMPO–OH adducts formed through the participation of DMPO and hydroxyl radicals, which are produced from primary persistent radicals generated in CA reactor. The use of DMPO is convenient and a reliable conclusion can be reached in accurate experimental settings. In this context, we compared the generation of hydroxyl radicals from both MCP 230 EPFRs and EPFRs collected from HL pyrolysis in the CA reactor under aerated and nonaerated conditions of the respective solutions.^31,61^ The samples investigated showed the typical 4-line split (1:2:2:1) EPR spectrum characteristic to the DMPO–OH adducts.^28,31,61,62^ All the samples were measured 10 min after adding DMPO and monitored for a total time of 180 min. To analyze the resulting spectra, the normalized double integration (DI/N) of the EPR spectra for all samples was standardized to the DPPH reference and plotted against time.

### 4.1 Effect of Aeration over Spin Trapping Experiments in the Presence of EPFRs

#### 4.1.1 Aeration of the Sample Containing Fenton Reaction Components (Ferrous Ammonium Sulfate and H_2_O_2_)

Decades ago, Haber and Weiss discovered that the highly reactive hydroxyl radical (HO^•^) could be generated from an interaction between superoxide (O_2_^•–^) and hydrogen peroxide (H_2_O_2_)^63^
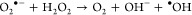
R1

Haber and Weiss discussed also the need for a metal ion catalyst and illustrated that the net reaction R1 can be broken down into two chemical reactions R2 and (R3). Iron-catalyzed Haber-Wise reaction R1 makes use of Fenton chemistry, which is now considered to be the major mechanism by which the highly reactive hydroxyl radical is generated in biological systems, reaction R3.^64^
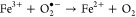
R2
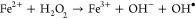
R3

**Figure 3:**
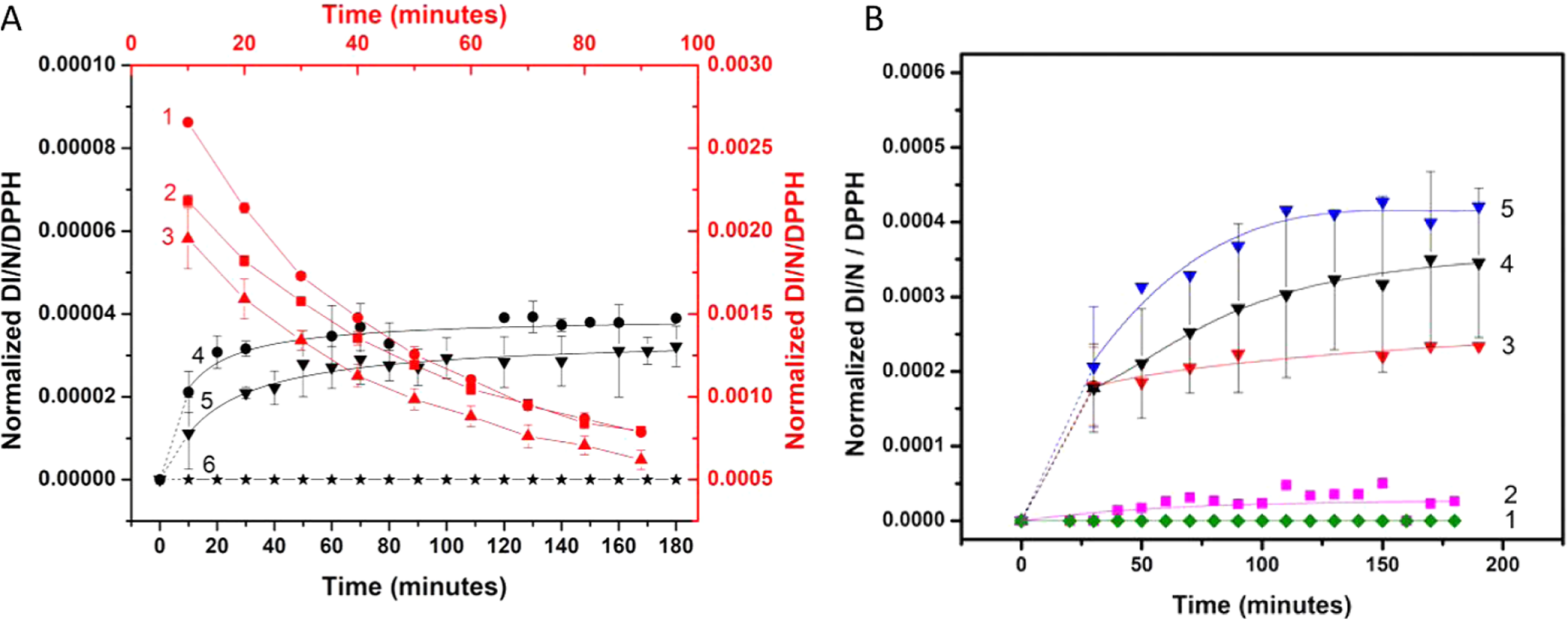
Time dependence of EPR signal intensity of DMPO–OH adducts from (A) Fenton reaction (red line 1—after air bubbling, line 2—no bubbling, and line 3 after N2 bubbling); from solution containing MCP230 EPFRs, black line 4 after air bubbling, line 5 for catalyst only, and line 6 for silica only. (B) Reference solutions [line 1—deionized water + Cambridge filter (CF) + DMPO, no bubbling; line 2—buffer + CF + DMPO, no bubbling] and the solutions containing EPFRs derived from lignin pyrolysis; line 3—after air bubbling, line 4—no bubbling, line 5—bubbling by nitrogen.

The key role oxygen plays in the Fenton reaction can be observed in Figure 3A. Partially removing dissolved oxygen from the Fenton solution by bubbling it with nitrogen slows the generation of DMPO–OH (line 3) while aerating the solution increases the yield of DMPO–OH (line 1). In both cases, the intensity of the spin adduct decreases over time due to the consumption of oxygen.^65^

It is noteworthy that the production of DMPO–OH in air-bubbled samples in the Fenton reaction gets a higher decline toward 40 min up to the end of the experiment where air bubbled sample gets closer to the no-bubbling sample (line 2) until they overlap at nearly 65 min. These observations as well as recent studies^66−68^ confirm the positive role of oxygen in Fenton reactions; it provides a more oxidative environment for Fe (II) and faster reaction rates to produce ROS. Removing oxygen generates less H_2_O_2_ in the presence of other inert gases such as N_2_, Ar, or He.^69−71^ Nevertheless, the somewhat controversial impact of aerating Fenton-like solutions on OH radical generation is briefly discussed in the Supporting Information (Section 7.1).

#### 4.1.2 Spin Trapping from the Solution Containing EPFRs Generated from MCP and Lignin: The Effect of Aeration

The effect of aeration of the samples containing EPFRs in terms of generating more hydroxyl radicals was demonstrated in a number of early publications.^28,61,62,72^ Aerated samples proved to have a much higher quantity of hydroxyl radical compared to the nonaerated sample, containing EPFRs generated from MCP at 230 °C,^28^ Supporting Information, Figure S11, section 6.2. This phenomenon was validated in current work at aeration of the solution containing 1.25 mg/mL concentration of MCP230 EPFRs particulates at 1.37 × 10^17^ spins g^–1^ (line 4, Figure 3A) in comparison with a nonaerated solution containing only the catalyst, CuO/SiO_2_ (line 5, Figure 3A). As documented in previous publications, the high yields of DMPO–OH adduct on catalyst surfaces noncontaining EPFRs (line 5, Figure 3A) are due to known artifacts such as nonradical hydroxylation of DMPO by catalytic surfaces.^61,73−75^

Dramatically different results were observed from the spin trapping experiments in the presence of EPFRs produced from pyrolysis of HL in the CA reactor: the aeration of solution inhibits the formation of DMPO–OH meaning generation of hydroxyl radicals is suppressed in the presence of oxygen, (Figure 3B). Purging dissolved oxygen by bubbling of the solution by nitrogen promotes formation of hydroxyl radicals and hence increases the intensity of DMPO–OH adduct.

This phenomenon can be explained by the formation of oxygen-centered radicals during lignin pyrolysis, as shown in Figure 2A,B. The radical species presented in Figure 2B are assumed to be o-semiquinone radicals (o-SQ)^76^ stabilized in the polyphenolic lignin matrix. The biological importance of SQ radicals has been at the center of attention for many decades, from tobacco research^53^ to the present day.^50,51,76^ SQ radicals are responsible for reducing oxygen to superoxide and hydrogen peroxide (reaction R4).^53,54^ SQ radicals or quinones (Q) normally involved in physiological processes (ref Supporting Information, Figure S12, section 6.3) play a role as catalysts of the Haber–Weiss reaction R5 in the biological generation of hydroxyl radicals. In fact, in metal metal-free environment quinones shuttle electrons to H_2_O_2_ by initiating a reductive homolytic cleavage of H_2_O_2_ giving rise to the formation of OH radicals, reaction R5. In other words, reductive homolytic cleavage of H_2_O_2_ is observed to depend not only on the presence of transition metals but also on quinones in the reaction medium, as directly shown in ref (77). Note that metal-independent production of hydroxyl radicals was advocated in ref (78) by Zhu et al.
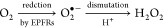
R4
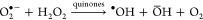
R5

**Scheme 3:**
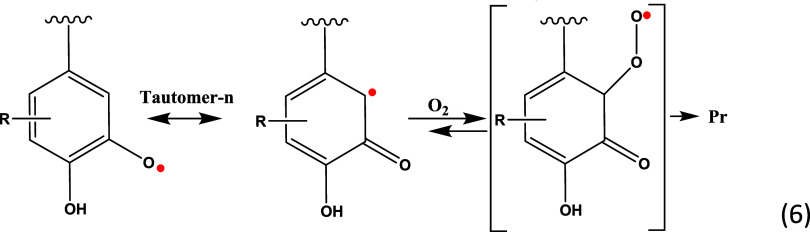
Removal of SQ Radicals by Oxygen

Therefore, any competitive reaction toward reaction R4, for example, removal of SQ radicals by reaction R6, Scheme 3 at increasing concentration of O_2_ (aeration) inhibits process generation of hydroxyl radicals and hence, suppresses the formation of superoxide radicals and therefore, H_2_O_2_ through reaction R4. Additionally, other types of stabilized carbon-centered radicals after pyrolysis of HL^8^ can remove oxygen from the medium, thereby negatively affecting the reaction R4. All these factors reduce the formation of quinones in aerated samples which leads to a slowing of catalytic activity in reaction R5. A recent publication has reported similar behavior in aerated solutions of rhodamine B (RhB).^79^ Specifically, the removal efficiencies of RhB in the Fenton system without O_2_ were found to be higher than those in the Fenton system with O_2_ at various H_2_O_2_ concentrations. This phenomenon is not readily apparent in spin-trapping experiments for MCP230 EPFRs. While dissolved oxygen may influence the concentration of surface-associated EPFRs (as observed with EPFRs stabilized on a lignin matrix), the exogenous Fenton reaction^31^ involving low-valence-state transition metals remains a source of superoxide radicals generation (Supporting Information, section 6.4), and consequently, H_2_O_2_ production increases with higher oxygen concentration.

## 5 Conclusions

The primary process of depolymerization of lignin dispersed into a gas phase has been examined in fast-flow, continuous atomization (CA) reactor. It was documented that the primary processes of lignin depolymerization occur via the fragmentation of lignin macromolecules, producing intermediate oligomer radicals and neutral oligomer fragments. These intermediates form without producing major biofuel components, such as phenolics, exclusively in the gas phase. Specific attention was focused on the behavior of intermediate environmentally persistent free radicals (EPFRs) derived from homogeneous–heterogeneous pyrolysis of lignin in a CA reactor. The biological activity of these EPFRs as OH radical generators in an aqueous environment has been demonstrated through spin-trapping experiments combined with EPR spectroscopy.

The addition of a highly hydroxylated CuO/SiO_2_ catalyst (5% CuO) at a concentration of 1–3% (relative to an initial lignin concentration of 1 g/L in a 9:1 acetone mixture) did not significantly affect EPFRs yields. However, a trend of increasing yields was observed with higher catalyst concentrations, particularly at 5%. A mechanistic scheme for the formation of CuO-surface-associated EPFRs is discussed.

## Supporting Information Available

Additional experimental details, and methods; ESI MS analysis of initial and pyrolyzed lignin; gas-phase exposure chamber; EPR spectra of EPFRs; Doehlert design, details; easySpin code; spin trapping, details; Redox cycling of semiquinone radicals (PDF)

## Nomenclature

LSU: Louisiana State University
HL: Hydrolytic lignin
EPFRs: environmentally persistent free radicals
EPR: electron paramagnetic resonance
LPHP: laser-powered homogeneous pyrolysis
CA: continuous atomization
ROS: reactive oxygen species
DIW: deionized water
RSM: response surface methodology (Doehlert’s design)
ESI MS: electrospray ionization mass spectrometry
HPLC: high-performance liquid chromatography
CFA: coniferyl alcohol
BDE: bond dissociation energy
GGE: guaiacylglycerol-β-guaiacyl ether
SQ: semiquinone radical
DMPO: 5,5-dimethyl-1-pyrroline-*N*-oxide
CF: Cambridge filter
