# Supplementary material for: Primary Processes of Depolymerization of Lignin Dispersed into Gas Phase
Source: Energy Fuels. 2025 May 5;39(19):8954–63. doi: 10.1021/acs.energyfuels.4c06278 (PMC12086844; doi:10.1021/acs.energyfuels.4c06278)
Supplement: Supplementary file 1 — ef4c06278_si_001.pdf [file ef4c06278_si_001.pdf]

Supporting Information

Primary Processes of Depolymerization of Lignin Dispersed into Gas Phase

Marwan Y. Rezk[1], Mohamad Barekati-Goudarzi[1], Divine Nde[2], Dorin Boldor*[1]

Slawomir Lomnicki[3], Stephania Cormier[4], Lavrent Khachatryan*[2]

[1] Department of Biological and Agricultural Engineering, Louisiana State University and LSU AgCenter, Baton Rouge, United States, 70803,

[2] Department of Chemistry, Louisiana State University, Baton Rouge, United States, 70803,

[3] Department of Environmental Sciences, Louisiana State University, Baton Rouge, Louisiana 70803, USA

[4] Department of Biological Sciences, LSU Superfund Research Program and Pennington Biomedical Research Center, Baton Rouge, Louisiana 70808, USA

1. Methods

CA reactor

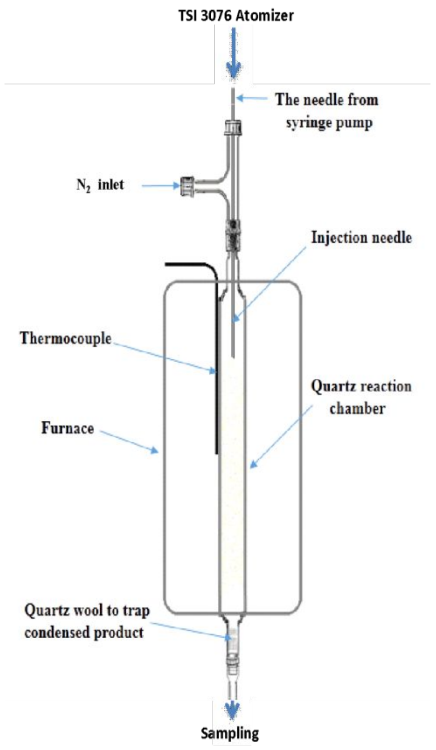

Fig. S1. *Continuous atomization (CA) fast flow reactor coupled with TSI 3076 Atomizer.*

EPR Measurements: Bruker EMX-2]0/2.7 EPR spectrometer (X-band) with dual cavities was used to record EPR spectra generated from lignin pyrolysis. The settings for spectra acquisition were kept constant unless required and noted. Spectra was acquired with modulation of 100 kHz, microwave frequency of 9.72 GHz. Typically, sweep width was 100 G, microwave power was 2mW, modulation amplitude, 4.0 G (1G for spin trapping experiments), time constant, 40.96 ms, and sweep time of 167.777s. To calculate the concentration following acquisition 2,2-diphenyl-1-picrylhydrazyl (DPPH) standard was used as a reference.

ESI-TOF-MS analysis: The HL pyrolysis samples were dissolved in acetonitrile (ACN)/water with 5% formic acid (FA) and run in positive mode ionization with a capillary voltage of 4000v. Drying gas (nitrogen) temperature was 325°C delivered at 10 l/min and the fragmentor voltage was set to 150 **v. No** LC column was used for sample delivery; only flow through injection was utilized (direct injection from LC to mass spectrometer). Mobile phases used were A: 30% LCMS grade water with 0.1% formic acid and B: 70% LCMS grade acetonitrile with 0.1% formic acid with a flow rate of 0.4ml/min.

Spin Trapping:

This section involves Fenton reaction components (320 µL H_2O that was deoxygenated beforehand) + 30 µL of 3M DMPO + 20 µL 30 % H_2O_2 + 30 µL of 150 µM ferrous ammonium sulfate sol to give a total of 400 µL), EPFRs from MCP, and lignin. Each of the previously mentioned 3 experiment types was tested under no bubbling, under nitrogen, or oxygen bubbling for the PBS buffer solution. To prepare the particles solution; the particles were suspended in buffer solution followed by vortex and sonication for 5 minutes each, respectively. The spin trapping experiments for MCP/lignin were done by adding a total of 10 µL from a previously prepared stock solution of 3M DMPO to 10 µL of lignin products solution as well as 180 µL of the previously prepared buffer solution. For MCP samples, the EPFRs were obtained in glass sealed vials to keep free radicals active, the protocol for MCP free radicals' generation can be found elsewhere [1]. The final solution was vigorously shaken at the dark using Vortex Genie 2. EPR capillary tubes were used to draw 20 µL of the solution then sealed with end sealant (Fisher, USA). The capillary tube was scanned using EPR after inserting it into the 4 mm EPR tube.

2. Detection of Oligomers from HL pyrolysis in CA reactor

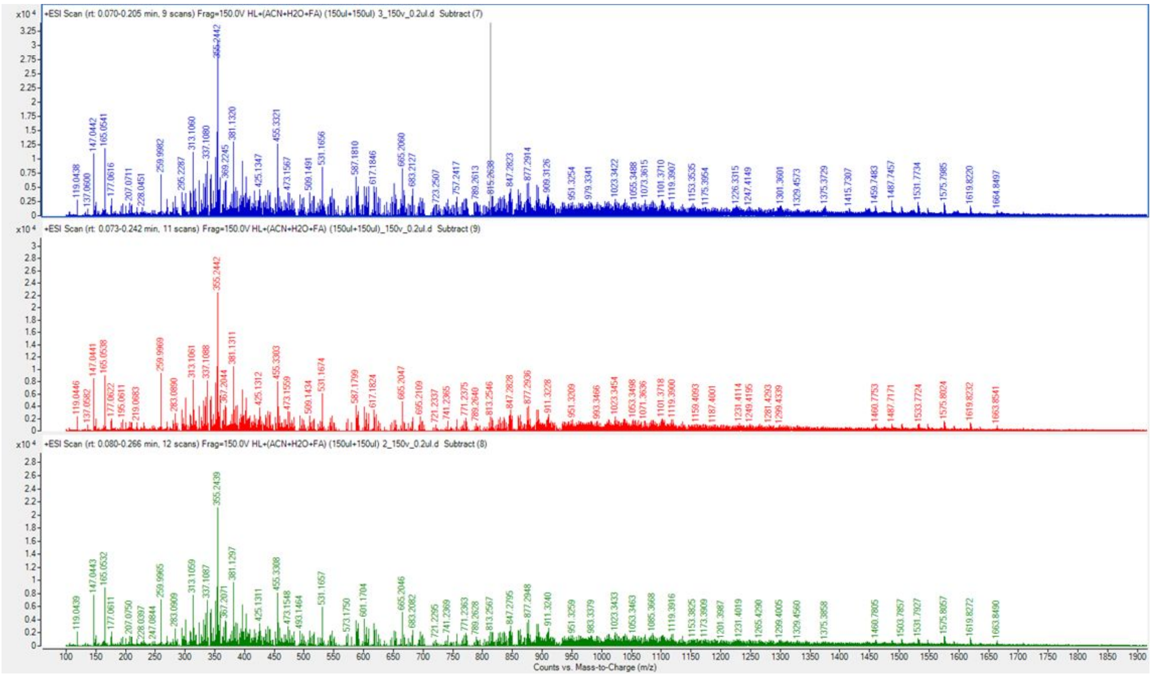

Fig. S2. ESI MS analysis - Triplicate injection of HL dissolved in ACN/water/formic acid solution

ESI-TOF-MS analysis of initial HL: In Fig.S1 and Table S1 triplicate injections for pure initial lignin is represented.

Table S1. Area percent of grouped oligomers in the initial HL after triplicate injections (HL1-HL3)

	100-250	250-400	400-550	550-700	700-850	850-1000	1000-1150	1150-1300	1300-1600
HL 1	13	21.40	13.20	15.70	12.90	7.40	5.70	4.60	6.10
HL 2	8.6	20.6	14	16.5	14.1	7.9	6.3	5.2	6.8
HL 3	7.5	20.8	13.7	17.1	13.9	7.9	6.5	5.3	7.4

Temperature Dependence of the yields of Oligomers: Temperature dependence of the yields of oligomers from pyrolysis of HL dispersed in CA reactor was investigated in the range 450 to 550 C. All experiments were at least duplicated to ensure reproducible results. The temperature dependence of the area (in percentage) of grouped oligomers from HL pyrolysis in CA reactor is summarized in **Figure S3**.

As it can be seen, there are not large changes between the yields of different oligomers with

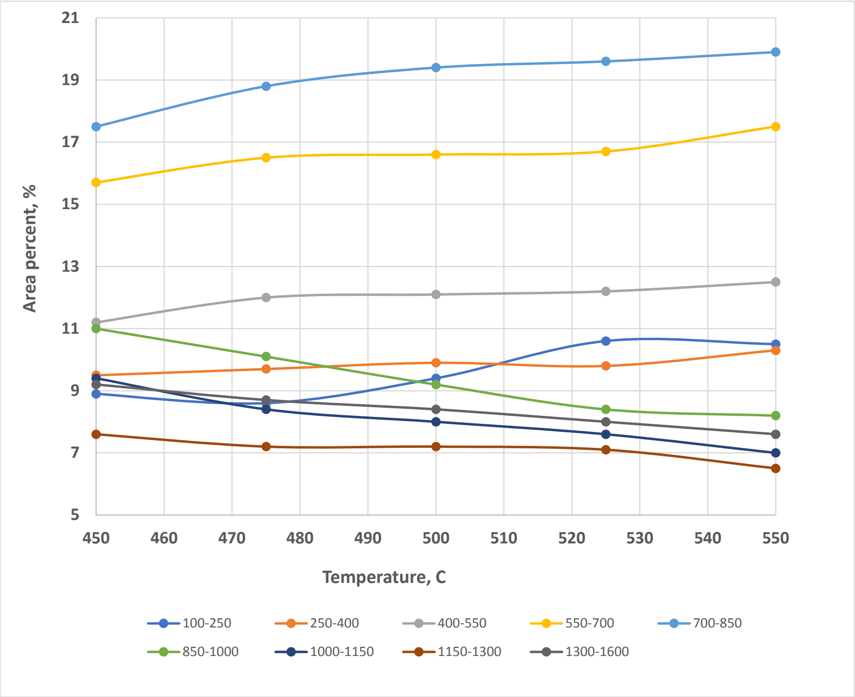

Fig. S3. The area percentage of the grouped oligomer products from pyrolysis of HL in CA reactor at different temperatures.

increasing temperature. The concentration (the area percent) of some groups of oligomers slowly is increasing (100-250; 550-700; 700-850) or decreasing (850-1000; 1000-1050; 1300-1600). The concentration of a few groups of oligomers stays nearly unchanged (250-400; 400-550;1150-1300).

Due to different trends of the temperature dependence of the concentration of oligomers from HL pyrolysis in CA reactor a redistribution of each mass track in the ESI MS spectra relative to the initial lignin may occur. Similar behavior has also been detected from pyrolysis of coniferyl alcohol, CFA in CA reactor [2].

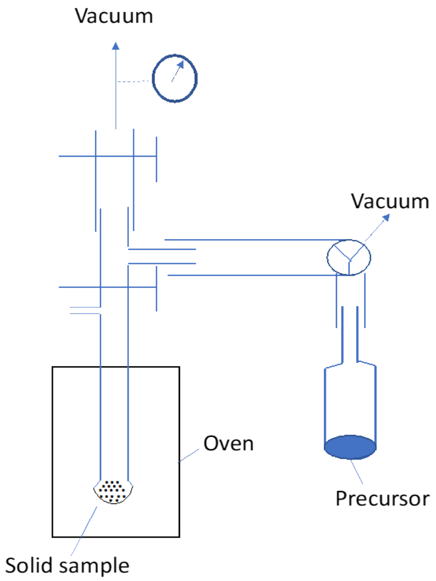

Fig. S4. Gas-phase exposure chamber

3. Gas-phase exposure chamber for generation of EPFRs [3].

The 5% CuO/SiO_2 catalyst prepared according to the procedure described in Refs [3] [4] was exposed to saturated

vapors of MCP (or CT) at 230 °C under vacuum (10^{-2} torr) for 5 min repeatedly a few times, **Fig. S4**. Then the exposure chamber was allowed to cool down to 50 °C under vacuum and the particulate was subjected to EPR analysis (X band EPR, Bruker EMX-20/2.7).

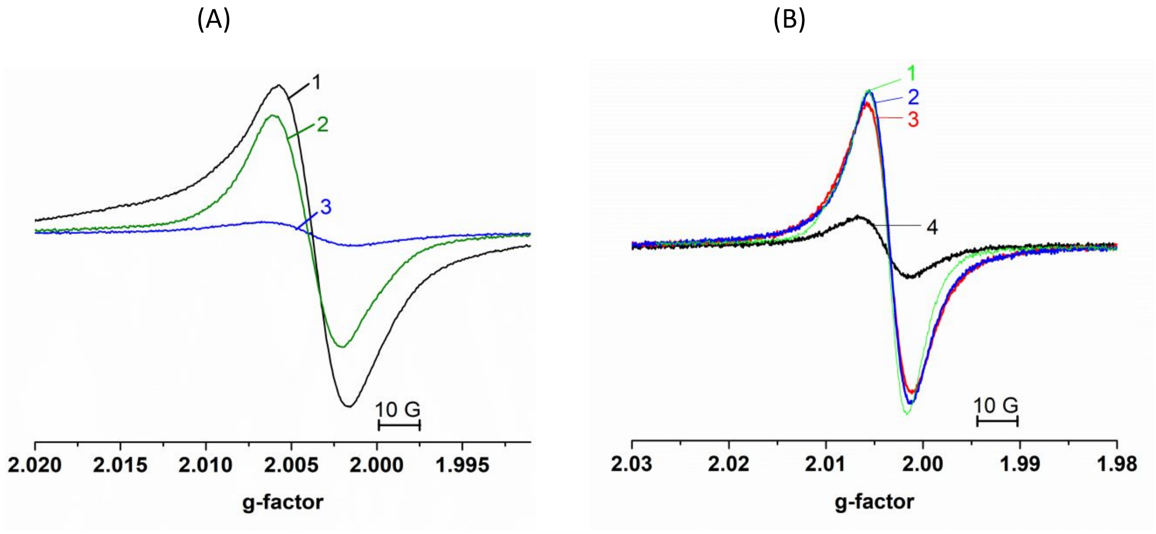

Fig. S5. EPR spectra for (A) 1 - CT230 EPFRs (g-factor =2.0038, ΔH = 7.27 G); 2 – MCP230 EPFRs (g-factor =2.0041, ΔH = 7.1 G); 3 – EPFRs from 1% HL mixed with CuO/SiO_2 and heated at 230 °C (g-factor =2.0040, ΔH = 9.0 G).
(B) EPFRs after heating of hydrolytic lignin (1 %, w) in the presence of highly hydroxylated CuO/SiO_2 catalyst; (1) at 486 °C from CA reactor (green line – g-factor =2.0036, ΔH = 7.1 G), (2) at 290 °C (blue line - g-factor =2.0035, ΔH = 7.6 G), (3) at 270 °C (red line - g-factor =2.0035, ΔH = 8.1 G), at 230 °C (black line – g-factor =2.0039, ΔH = 8.9 G).

4. Doehlert design [5]: In this study, a two-factor Doehlert design was performed that resulted in the validation of second order mathematical models that can predict and optimize free radicals' concentration for lignin pyrolysis via continuous atomization. The planned experimental design parameters and conditions along with the actual experimental results are shown in **Table S3**.

Table S3. Experimental design conditions and resulting EPFRs conc. for hydrolytic lignin pyrolysis via CA reactor.

Coded values		Real values		
X1	X2	Residence time (s)	Reactor temperature (°C)	EPFRs conc. (spins g^{-1}) (Y)
0	0	0.35	475	1.44E+18
0	0	0.35	475	1.73E+18
1	0	0.6	475	2.22E+18

1	0	0.6	475	2.67E+18
0.5	0.866	0.475	550	7.65E+17
0.5	0.866	0.475	550	6.00E+17
-0.5	-0.866	0.225	400	7.40E+17
-0.5	-0.866	0.225	400	1.03E+18
0.5	-0.866	0.475	400	5.80E+17
0.5	-0.866	0.475	400	1.22E+18
-0.5	0.866	0.225	550	2.18E+18

The real values are related to the coded values using the expression below:

$U_0 = (A_{min} + A_{max})/2$ where A are real values of the variable, Uo is center point,

$\Delta U = (A_{max} - U_0)/B_{max}$ where B are the coded values, ΔU is increment

Residence time = $U_0+(B*\Delta U)$; where $-1 < B < 1$ and $0.225\ s < A < 0.6\ s$

Pyrolysis temperature = $U_0+ +(B*\Delta U)$ where $-0.866 < B < 0.866$ and $400\ °C < A < 550\ °C$.

R^2 and P-value of a model are used to describe its validity and the significance of the parameters on the process or phenomenon under study. Data with a P-value of less than 0.05 is considered significant on the response. In addition, the value of R^2 is a statistical estimate of how a dependent variable is strongly affected by change in independent variables. In this work we used 3 regression metrics to enhance the credibility of the Doehlert design. The first was R^2 which was obtained from regression analysis following the second order polynomial equation selected to model this process (Equation 1 in the main text). The second was residual analysis expressed as the

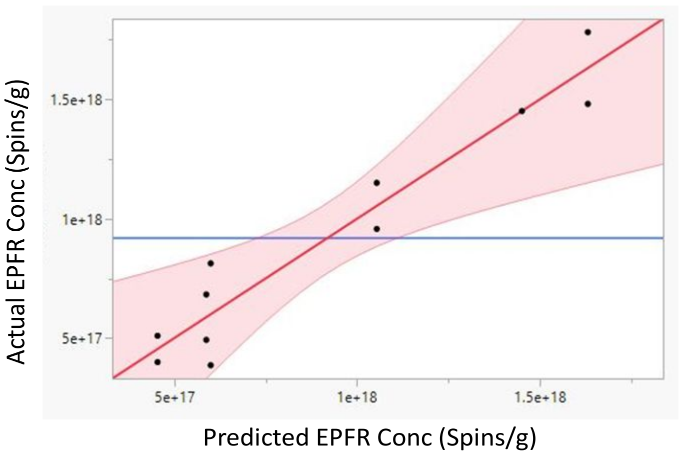

Figure S6: Plot of actual concentration of EPFRs vs. predicted concentration shows uniform distribution of points around the diagonal line which validates the model

mean absolute error of deviation (AED) of the calculated EPFR concentration from the actual values which were estimated from equation equation S1;

$$AED = 100\sum_{1}^{n}\left|\frac{Actual - Estimated}{Estimated}\right|/n, \qquad \text{Equation S1}$$

where n is the number of experimental runs carried out.

The third was a plot of the predicted or calculated values vs. actual values, Fig. S6. For a model to be considered valid, at least one of the regression matrices should satisfy the following conditions $R^2 > 0.70$, AED < 10% and a uniform distribution of points around the diagonal of the plot of predicted vs. experimental values [6]. In current work the R^2 value for the EPFRs concentration was 0.92, at 17% of AED and a uniform and equitable distribution of points around the diagonal Fig. S6. These metrics indicated that the selected second order equation was credible to describe the lignin depolymerization process in the continuous atomization reactor. Note that a 17% error margin in this work was considered acceptable given the very small quantities of depolymerization products that were collected at the end of each experiment (0.6 – 0.8 mg). Small changes in repetition could give rise to large variations in the concentration of EPFRs expressed in spins/g.

The model's P values and the R^2 for the EPFRs concentration were 0.007 and 0.92 respectively, indicating that EPFR concentration were significantly dependent on the reaction temperature and residence time. R^2 value of 0.92 indicates that 92% of the dependence of EPFR concentration on temperature and residence time can be explained by the model.

Table S4: Regression coefficients, P-value and R^2 value for EPFRs conc.

	Coefficients	P-value
β0	1.05E+18	0.000527
β1	-4.9E+17	0.021696
β2	2.07E+17	0.0616
β11	1.07E+18	0.006799

β22	-7.3E+17	0.008522
β12	-5.8E+17	0.019866
R^2	0.92	
P-value	0.0079	

From the p-value of **Table S4**, it can be observed that there is a significant influence of residence time (β1) and the interaction between temperature and residence time(β12) on the concentration of EPFRs. Similarly, we see significant effects of β11 and β22. However, these effects only account for the curvature of the quadratic equation behavior. Response surface plot **Fig.S7**. indicate that more radicals were generated at high temperatures for a short residence time or at lower temperatures within higher residence times. However, at higher temperatures and prolonged residence times the EPFR concentration dropped dramatically as depicted in the negative coefficient of the interaction between residence time and temperature in **Table S4**. This negative coefficient indicates that the interaction coefficient of temperature and residence time has an inverse relation with the EPFRs concentration. Increasing concentration of radicals at high temperatures within short residence times can be explained by fission of β-O-4 bonds which are then released as fragments into the gas phase. Barekati et all reported a similar phenomenon of increase

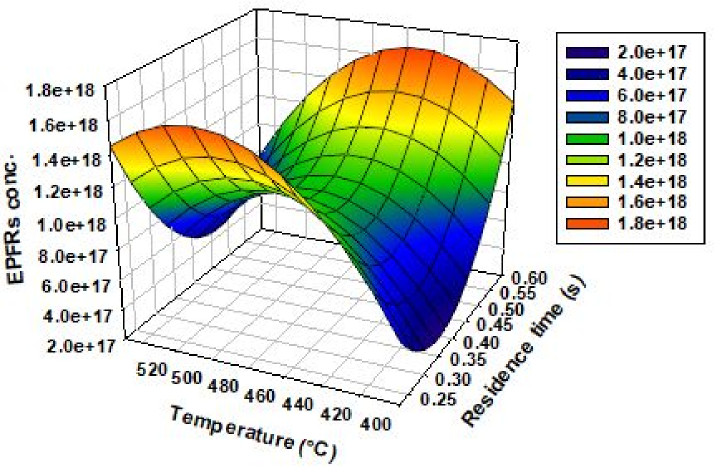

Fig.S7. EPFRs concentrations are profoundly affected by interaction of temperature and residence time. Secondary reactions are responsible for the significant decrease at prolonged residence time and higher temperatures.

generation of EPFRS from the from lignin pyrolysis in vacuum and trapped cryogenically at 77 K with an increase in temperature and proposed that the phenomenon could additionally be linked to the formation of oxygen-centered radicals easily formed from nascent lignin molecules in the gas phase [7]. The reversed but not surprising phenomenon observed at high temperatures with prolonged heating is likely due to change of the mechanism of depolymerization of HL occurring in the reactor as reaction progresses. Zhang et al. has given [8] a detailed analysis of the radical cleavage of specific interunit linkages (β-O-4, α-O-4, β-5, β-β) and the subsequent reactions of the formed radicals.

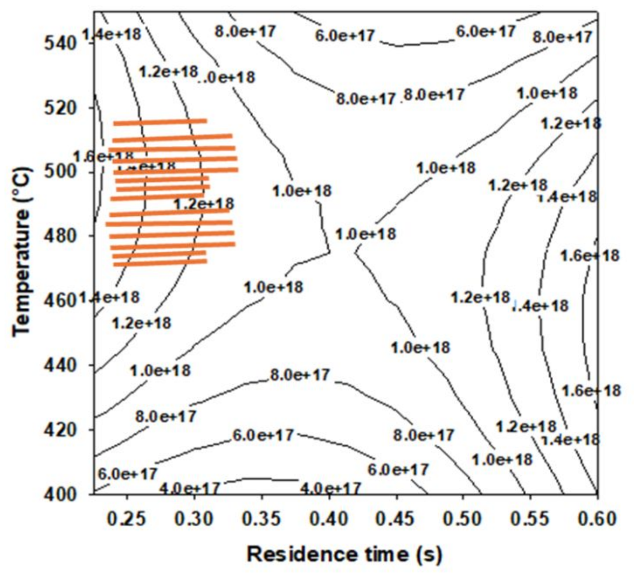

Fig. S8. Contour plots for the CA of lignin showing that optimum depolymerization conditions were in the range 460-520 °C and 0.2-0.35s for temperature and residence time respectively.

A contour plot of the depolymerization process in **Fig.S8** depicts the optimum processing zone of 460-520 °C and 0.2-0.35s for temperature and residence time respectively. The residence time 0.3sec at 486 °C was retained for generating EPFRs which were subsequently used for spin trapping experiments. The selected conditions were verified by carrying out separate experiments and comparing the EPFR concentration to that calculated from the validated equation (Fig.S6). The aforementioned optimal conditions yielded a concentration of free radicals of 2.40E+18 spins/g , g-factor of 2.0034, and ΔHp-p = 7.137 gauss. These optimal values are in good agreement with previous work in literature using hydrolytic lignin pyrolysis in gas phase[19].

The optimal conditions determined using Doehlert's design were applied in subsequent spin-trapping experiments (Section 4 in the main text) to achieve high yields of free radicals, enabling better characterization of the biological consequences of these precursors in ROS production.

5. Lignin model structure , β-O-4 model dimer (guaiacylglycerol-β-guaiacyl ether, GGE)[9].

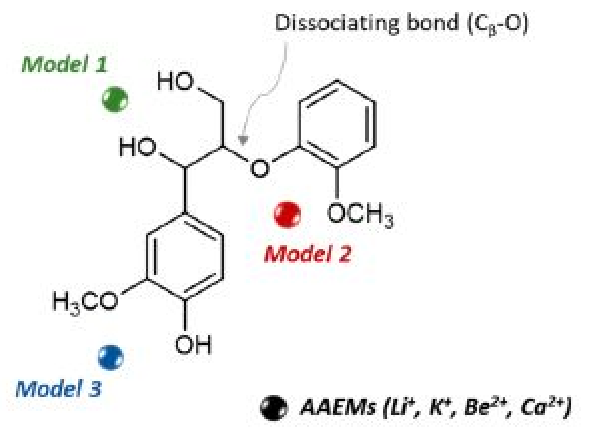

Fig.S9. The binding sites of AAMEs (alkali and alkaline earth metals) on GGE. Model 1: O(Cα)–M–O(Cγ), model 2: O(Cβ)–M–O(methoxy), and model 3:(hydroxyl)–M–O(methoxy).

EasySpin code for spectral simulation of HF EPR spectrum of PFRs derived from HL pyrolysis in CA reactor [10].

```
[B, spc] = textread('5K413dwM25_50kHz_0.5mTs.txt');%The text file of HF EPR spectrum
B = B/10;  % G -> mT
plot(B,spc);

Sys1.g = [2.0046 2.0033 2.0024];%Bahrle data 11.14.24
Sys1.lwpp = 13.0;  % mT, was 17.7
Sys1.weight = 0.97;%0.97
Sys2.g = 2.003;%2.003
 Sys2.lwpp = 5.30;  % mT was 6
Sys2.weight = 0.09;% was 0.09

Exp.mwFreq = 413;  % GHz
```

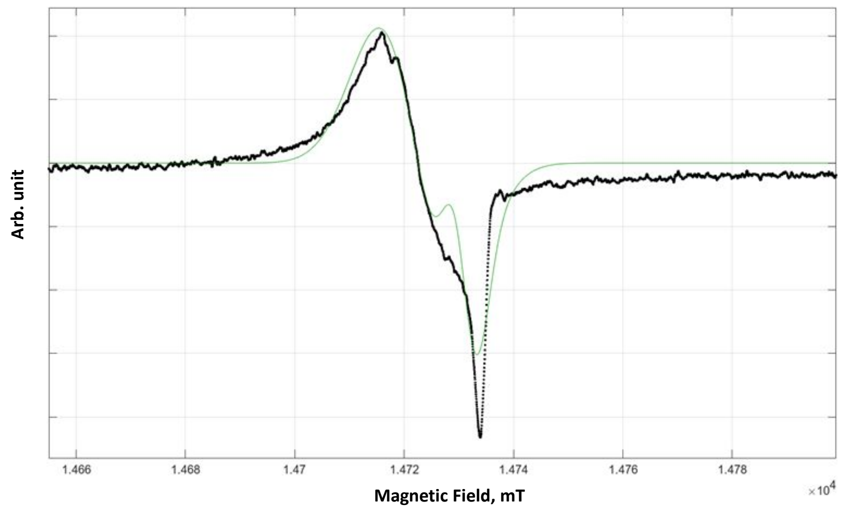

Fig. S10. EasySpin spectral simulation (green line) of HF EPR spectrum of PFRs derived from HL pyrolysis in CA reactor (black line).

```
Exp.Range = [14655
14799];  % mT
Exp.Temperature =
5.0;  % temperature,
in kelvin

Sys1.g = [2.0042
2.0033 2.0024];% was
1.98
Sys2.g = 2.0015;
Sys2.lwpp = 5.30;

Sys2.weight =
0.1;%was 0.1
Sys = {Sys1,Sys2};

Vary1.g = [0.0012
0.001 0.001];%was
0.0012 0.001 0.001
Vary2.g = 0.0016;
Vary2.lwpp = 0.002;
Vary2.weight = 0.003;%was 0.003
%Vary.lwpp = 2;

Vary = {Vary1,Vary2};

% Call the fitting function
SimOpt.Method = 'perturb';
FitOpt.Method = 'simplex int'; % simplex algorithm, integrals of spectra
esfit(spc,@pepper,{Sys,Exp,SimOpt},{Vary},FitOpt);
```

6.0. Spin trapping

6. 1. Fenton reaction: The role of oxygen in Fenton-like reactions has been under debate in literature over the years. The faster degradation of 4-chlorophenol by Fenton system in the presence of oxygen was acquired at higher concentration of oxygen compared to those obtained in at nitrogen environment[11]. A study for the degradation of reactive black 5 in a Fenton system under varying initial dissolved oxygen concentration indicated that the removal efficiency increased with decreasing the oxygen concentration[12]. This negative effect was ascribed to the competition between O_2 and reagents for organoradicals. Other research also indicated similar negative effect that was ascribed to the competition between ferrioxolate and O_2 for organoradicals[13]. Huston et al explained the negative effect by the oxygen’s reaction with

intermediate free radicals such as $C_2O_4^{\cdot -}$ and/or $CO_2^{\cdot}$ that prevents the degradation/conversion reaction[14].

6.2. Spin Trapping. Aeration of the sample MCP230 EPFRs [15]: The results of non-

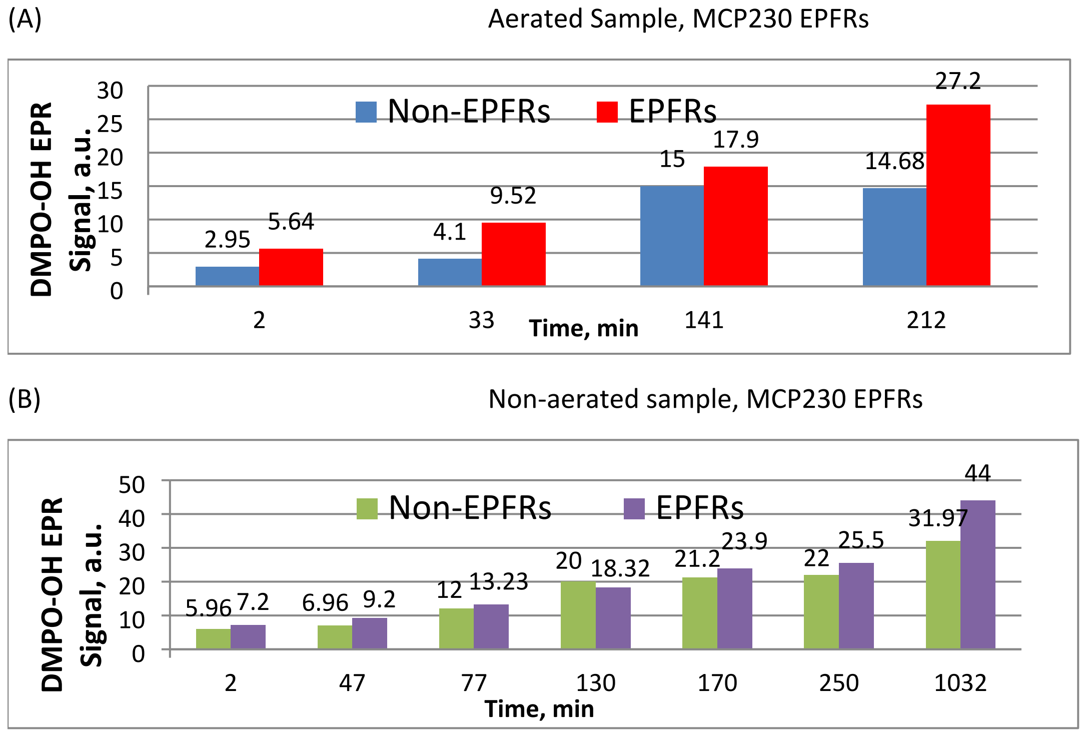

Fig.S11. The effect of aeration of the samples containing MCP230 EPFRs in generation of DMPO-OH adducts: (A) -aerated and (B)- non-aerated solutions in comparison with the solution containing catalyst only.

aeration/aeration on both non-EPFR (control) and EPFR containing particles solutions are presented in **Fig. S11, A and B**. The difference in the DMPO-OH adduct spectral intensity for the sample and control solutions increased with time, most notably at incubation times > 150 min. This difference was larger and occurred at earlier times for the aerated solution. For instance, the difference in the non-aerated solution was only ~ 50 % at 1055 min for the non-aerated solution but was ~100 % for the aerated solution at only 220 min. These results confirm involvement of O_2 in the redox cycle generating OH radical[15].

6.3. Redox cycling of semiquinone radicals (SQ), Fig. S12 [16 17]:

$$Q + QH_2 \leftrightarrow 2(\bullet QH) \quad 1)$$

$$\bullet QH + O_2 \rightarrow Q + O_2^{\bullet -} + H^+ \quad 2)$$

$$2O_2^{\bullet -} + 2H+ \rightarrow O_2 + H_2O_2 \quad 3)$$

$$H_2O_2 + Fe^{+2} \rightarrow HO\bullet + HO^- + Fe^{+3} \quad 4)$$

$$\bullet QH + Fe^{+3} \rightarrow Q + Fe^{+2} + H^+ \quad 5)$$

Scheme 1 +

(•QH) stands here for the principle radical semiquinone, (SQ)

Fig.S12. + A basic scheme for the role of SQ radicals for generation of H2O2 from tobacco research

Quinones may play a role as catalysts of the Haber-Weiss reaction (main text, reaction 5) in the biological generation of hydroxyl radicals which has been verified in ref. [18].

6.4. Exogeneous Fenton reaction [15]. Surface copper (Cu^{+1}) associated with EPFRs (abbreviated $R^{\bullet}$ below) generate superoxide radicals through complexation with oxygen.

$$\underline{R^{\bullet}\text{---}Cu^{+1}} + O_2 \rightarrow \underline{R-Cu^{+2}} \text{ -- } O_2^{\bullet -}$$

$$\underline{R-Cu^{+2}} \text{ -- } O_2^{\bullet -} \rightarrow \underline{R-Cu^{+2}} + O_2^{\bullet -}$$

References

(1) Khachatryan, L.; McFerrin, C. A.; Hall, R. W.; Dellinger, B. Environmentally Persistent Free Radicals (EPFRs). 3. Free versus Bound Hydroxyl Radicals in EPFR Aqueous Solutions. *Environmental Science & Technology* **2014**, *48* (16), 9220-9226. DOI: 10.1021/es501158r.

(2) Barekati-Goudarzi, M.; Khachatryan, L.; Boldor, D.; Xu, M. X.; Ruckenstein, E.; Asatryan, R. Radicals and molecular products from the gas-phase pyrolysis of lignin model compounds: Coniferyl alcohol, theory and experiment. *Journal of Analytical and Applied Pyrolysis* **2022**, *161*. DOI: ARTN 105413
10.1016/j.jaap.2021.105413.

(3) Lomnicki, S.; Truong, H.; Vejerano, E.; Dellinger, B. Copper oxide-based model of persistent free radical formation on combustion-derived particulate matter. *Environmental Science & Technology* **2008**, *42* (13), 4982-4988. DOI: 10.1021/es071708h.

(4) Khachatryan, L.; Rezk, M. Y.; Nde, D.; Hasan, F.; Lomnicki, S.; Boldor, D.; Cook, R.; Sprunger, P.; Hall, R.; Cormier, S. New Features of Laboratory-Generated EPFRs from 1,2-Dichlorobenzene (DCB) and 2-Monochlorophenol (MCP). *ACS Omega* **2024**, *9* (8), 9226-9235. DOI: 10.1021/acsomega.3c08271.

(5) Araujo, P.; Janagap, S. Doehlert uniform shell designs and chromatography. *Journal of Chromatography B* **2012**, 14-21.

(6) Bup, D. N.; Abi, C. F.; Tenin, D.; Kapseu, C.; Tchiegang, C. Optimisation of the Cooking Process of Sheanut Kernels (Vitellaria paradoxa Gaertn.) Using the Doehlert Experimental Design. *Food Bioprocess Tech* **2012**, *5* (1), 108-117. DOI: 10.1007/s11947-009-0274-z.

(7) Barekati-Goudarzi, M.; Boldor, D.; Marculescu, C.; Khachatryan, L. Peculiarities of Pyrolysis of Hydrolytic Lignin in Dispersed Gas Phase and in Solid State. *Energy & Fuels* **2017**, *31* (11), 12156-12167. DOI: 10.1021/acs.energyfuels.7b01842.

(8) Zhang, C. F.; Shen, X. J.; Jin, Y. C.; Cheng, J. L.; Cai, C.; Wang, F. Catalytic Strategies and Mechanism Analysis Orbiting the Center of Critical Intermediates in Lignin Depolymerization. *Chemical Reviews* **2023**, *123* (8), 4510-4601. DOI: 10.1021/acs.chemrev.2c00664.

(9) Jeong, K.; Jeong, H. J.; Lee, G.; Kim, S. H.; Kim, K. H.; Yoo, C. G. Catalytic Effect of Alkali and Alkaline Earth Metals in Lignin Pyrolysis: A Density Functional Theory Study. *Energy & Fuels* **2020**, *34* (8), 9734-9740. DOI: 10.1021/acs.energyfuels.0c01897.

(10) Stoll, S.; Schweiger, A. EasySpin, a comprehensive software package for spectral simulation and analysis in EPR. *J Magn Reson* **2006**, *178* (1), 42-55. DOI: 10.1016/j.jmr.2005.08.013.

(11) Du, Y.; Zhou, M.; Lei, L. The role of oxygen in the degradation of p-chlorophenol by Fenton system. *Journal of Hazardous Materials* **2007**, *139* (1), 108-115. DOI: https://doi.org/10.1016/j.jhazmat.2006.06.002.

(12) Aris, A.; Sharratt, P. N. Influence of Initial Dissolved Oxygen Concentration on Fenton's Reagent Degradation. *Environmental Technology* **2006**, *27* (10), 1153-1161. DOI: 10.1080/09593332708618729.

(13) Hislop, K. A.; Bolton, J. R. The Photochemical Generation of Hydroxyl Radicals in the UV−vis/Ferrioxalate/H2O2 System. *Environmental Science & Technology* **1999**, *33* (18), 3119-3126. DOI: 10.1021/es9810134.

(14) Huston, P. L.; Pignatello, J. J. Reduction of Perchloroalkanes by Ferrioxalate-Generated Carboxylate Radical Preceding Mineralization by the Photo-Fenton Reaction. *Environmental Science & Technology* **1996**, *30* (12), 3457-3463. DOI: 10.1021/es960091t.

(15) Khachatryan, L.; Vejerano, E.; Lomnicki, S.; Dellinger, B. Environmentally persistent free radicals (EPFRs). 1. Generation of reactive oxygen species in aqueous solutions. *Environ Sci Technol.* **2011**, *45* (19), 8559-8566.

(16) Pryor, W. A., Hales, B.J., Premovic, P.I., Church, D.F. The radicals in cigarette tar: Their nature and suggested physiological implications. *Science* **1983**, *220*, 425-427.
(17) Pryor, W. A., Prier, D.G., and Church, D.F. ESR Study of mainstream and sidestream cigarette smoke:Nature of free radicals in gas-phase smoke and in cigarette tar. *Environmental Health Perspectives* **1983**, *47*, 345-355.
(18) Nohl, H.; Jordan, W. The Involvement of Biological Quinones in the Formation of Hydroxyl Radicals Via the Haber-Weiss Reaction. *Bioorg Chem* **1987**, *15* (4), 374-382. DOI: Doi 10.1016/0045-2068(87)90034-4.
